# Advances in mild photothermal hydrogel-based therapies for bone and soft tissue injuries

**DOI:** 10.3389/fcell.2025.1696209

**Published:** 2025-09-26

**Authors:** Peng Na, Jing-Lun Jiang, Ren-Peng Lv, Fan Yang, Shi-Feng Li, Xian-Zhuo Chen

**Affiliations:** ^1^ Department of Burn and Plastic Surgery, Affiliated Hospital of North Sichuan Medical College, Nanchong, China; ^2^ Biotechnology Innovation Drug Application and Transformation Key Laboratory of Sichuan Province, North Sichuan Medical College, Nanchong, Sichuan, China

**Keywords:** hydrogels, mild photothermal effect, tissue repair, bone regeneration, multifunctional materials

## Abstract

Bone and soft tissue injuries resulting from trauma, metabolic disorders, and tumors pose a serious threat to public health, and their treatment faces numerous challenges, including infection, chronic inflammation, and impaired vascularization. Photothermal hydrogels, a new class of biomaterials, can sterilize tissues via photothermal therapy (PTT) and, through intelligent material design, exhibit multiple biological functions such as modulating the pathological microenvironment in bone and soft tissues. These properties have earned them a reputation as a “star material” in tissue engineering. However, excessive heating (above 50 °C) can cause irreversible thermal damage to tissues. Therefore, functional hydrogels that generate a mild photothermal effect (approximately 40 °C–45 °C) have recently become a research focus. This review provides a comprehensive overview of the types and fabrication strategies of photothermal agents used in mild photothermal hydrogels, systematically summarizes recent progress in their applications for bone and soft tissue injury repair, and delves into the underlying mechanisms by which they promote tissue regeneration. By summarizing current findings and outlining future perspectives on the use of mild photothermal hydrogels in modern regenerative medicine, we aim to advance the development of tissue engineering.

## 1 Introduction

Bone and soft tissue injuries caused by trauma, burns, tumors, and sports-related incidents have become increasingly common, representing a major global public health challenge (Jin et al., 2024). Functional hydrogels, one of the most promising therapeutic strategies, have been successfully applied in various tissue repair contexts (Enayati et al., 2024). Among these, photothermal hydrogels have emerged as an innovative material that has attracted extensive research attention in recent years due to their unique biological advantages (Xiao et al., 2025; Zhang R. et al., 2025).

Photothermal hydrogels primarily work by converting light energy into heat in response to near-infrared (NIR) irradiation (e.g., wavelengths of 808 nm or 1,064 nm), thereby triggering biological effects in local tissues. Based on the temperature range achieved, photothermal hydrogels can be categorized into mild photothermal, moderate photothermal, and high-temperature photothermal types (He et al., 2024). Compared to moderate and high-temperature photothermal hydrogels, mild photothermal hydrogels (operating at 40 °C–45 °C) have gained attention for causing less thermal damage to tissues and providing a broader therapeutic window. They are considered to have the following biological advantages: 1) enhanced blood circulation: mild heating can dilate microvasculature, improving local blood flow (Wu A. L. et al., 2025). 2) Activation of heat shock proteins (HSPs): For example, upregulation of HSP70 and HSP27 enhances cellular stress resistance and promotes tissue repair (Cheng et al., 2023; Jiang et al., 2024). 3) Regulation of cell behavior: Studies have shown that temperatures around 42 °C can promote fibroblast migration, osteoblast mineralization activity, and the angiogenic potential of endothelial cells (Gao et al., 2025; Zhang et al., 2022). 4) Simplified fabrication: Mild photothermal hydrogels can achieve stable temperature control with simpler processes, while also reducing the required concentration of photothermal agents and light intensity (He et al., 2024).

Given their broad application prospects in tissue engineering, this review delves into the construction strategies and fabrication methods of mild photothermal hydrogel systems. It provides a comprehensive analysis of their biomedical application scenarios and underlying mechanisms, summarizes the therapeutic limitations of mild photothermal effects, and outlines future development trajectories in the field of bone and soft tissue repair.

## 2 Construction strategies of mild photothermal hydrogels

### 2.1 Inorganic photothermal materials for mild photothermal hydrogels

**FIGURE 1:**
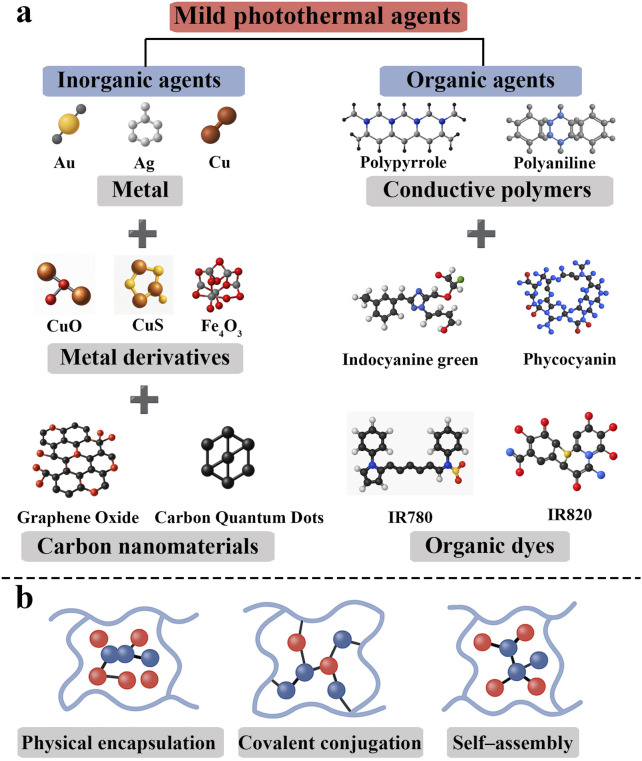
**(a)** Classification of mild photothermal agents; **(b)** Loading strategies for the agents.

Mild photothermal hydrogels generate a controlled photothermal effect by incorporating functional materials with high photothermal conversion efficiency; thus, the choice of photothermal agent is crucial for system design. The main photothermal agents currently in use can be classified into inorganic and organic materials (Li W. et al., 2023) (Figure 1a). Common inorganic photothermal materials include metallic nanomaterials and carbon-based materials.

#### 2.1.1 Metal-based inorganic photothermal agents

Noble-metal nanoparticles (e.g., Au, Ag, Cu) are well known to exhibit excellent photothermal performance owing to their localized surface plasmon resonance (LSPR) effects (Hu et al., 2024). By contrast, semiconductor-based photothermal agents operate via bandgap excitation rather than plasmonic effects. Absorbed photons in a semiconductor promote electrons across the bandgap, generating electron-hole pairs. Only semiconductors that are heavily doped or intrinsically defect-rich can approach the free-carrier densities of metals and thereby exhibit LSPR-like resonances (Liu et al., 2024). For this reason, metal-based nanoparticles are widely used as photothermal agents in applications such as cancer therapy, optical imaging, and solar-driven heat generation, where effective light-to-heat conversion is critical. In addition, these metal nanomaterials (especially noble metals like Au or Ag) are readily surface-functionalized that permit easy ligand attachment. For example, thiol-bearing molecules bind strongly to Au or Ag surfaces (forming metal–S bonds) (Cho et al., 2024), and original capping agents (e.g., citrate or CTAB) can be replaced by thiolated polymers, peptides or antibodies (ligand-exchange) (Duman et al., 2024). Likewise, charged ligands or biomolecules can adsorb electrostatically onto oppositely charged NP surfaces. These covalent (e.g., Au-S or amine-metal bonds) and noncovalent (electrostatic, hydrophobic) strategies allow stable conjugation of targeting ligands, drugs or imaging dyes to the nanoparticle surface (Cho et al., 2024).

Gold nanomaterials are among the most extensively studied photothermal agents. In recent studies, gold-based photothermal agents are often formed into photothermal composite hydrogels either by *in situ* generation or by pre-synthesis followed by incorporation into the hydrogel matrix. For example, one study directly mixed pre-made gold nanoparticles (AuNPs) into a calcium alginate hydrogel, upon irradiation with 808 nm near-infrared light (0.1–1 W/cm^2^) for several minutes, this system’s temperature rapidly rose to about 40 °C (Wu Y. et al., 2025). Silver nanoparticles (AgNPs) exhibit a similar LSPR photothermal effect to that of gold and also have inherent broad-spectrum antibacterial activity. Silver-loaded photothermal hydrogels are typically constructed by embedding or *in situ* generation of silver nanomaterials (Wang K. et al., 2025). Hao et al. reported a “carrier-free” silver nanoparticle hydrogel in which evenly dispersed AgNPs were generated *in situ* by the reduction of the natural polyphenol puerarin. Under 808 nm irradiation, the AgNPs produced a mild photothermal effect (∼45 °C) that quickly killed multidrug-resistant bacteria and continuously released silver ions to inhibit the regrowth of residual bacteria (Hao et al., 2025). Copper nanomaterials are cost-effective, have a broad antibacterial spectrum, and possess certain biological activities. Similar to gold and silver, copper nanomaterials also exhibit an LSPR effect. Lin and colleagues incorporated copper sulfide (CuS) nanoparticles into a hyaluronic acid hydrogel; under 808 nm irradiation, this material maintained a temperature of about 43 °C (Lin et al., 2021).

In summary, metallic nanomaterials (e.g., Au, Ag, Cu nanoparticles) offer strong NIR light absorption and highly efficient photothermal conversion due to their own chemical structural characteristics, and they can be readily functionalized for imaging or targeted delivery. However, these noble metal agents are non-biodegradable and tend to accumulate *in vivo*, raising concerns about long-term toxicity and clearance, which limits their clinical translation (Chen et al., 2024).

#### 2.1.2 Carbon-based inorganic photothermal agents

Carbon nanomaterials have high thermal stability and excellent thermal conductivity, and they are less prone to photothermal degradation. These materials also have a very large specific surface area, which allows them to carry growth factors, drugs, etc., and they can be functionalized through abundant surface groups (Cui et al., 2023; Liu et al., 2023).

Graphene and its derivatives (including graphene oxide [GO] and reduced graphene oxide [rGO]) have been widely studied due to their two-dimensional sheet structure and outstanding photothermal properties. A typical construction method is to introduce functionalized graphene into a hydrogel network to form a nanocomposite scaffold. For example, by grafting branched polyethyleneimine (BPEI) onto GO and then forming dynamic Schiff-base crosslinks with aldehyde-bearing polymers, a stable three-dimensional network was constructed. *In vitro* experiments showed that an osteogenic scaffold loaded with this functionalized could reach 43 °C after 3 min of 808 nm laser irradiation (Zhang et al., 2021). Carbon quantum dots (CDs) are zero-dimensional carbon nanoparticles smaller than 10 nm. In constructing mild photothermal hydrogels, a common strategy is to incorporate CDs into the hydrogel network. On one hand, the carboxyl and amino groups on the surface of CDs can undergo Schiff-base reactions with multi-aldehyde polymers, achieving dynamic covalent cross-linking (Sharma et al., 2022); on the other hand, CDs can be simply dispersed in a polymer matrix and held inside the gel through hydrogen bonding or electrostatic interactions (Lu et al., 2025). Carbon nanotubes (CNTs) are one-dimensional hollow tubular structures made of sp^2^-hybridized carbon atoms. They possess excellent mechanical, electrical, and thermal properties. Common construction methods include physical dispersion and surface functionalization. Physical dispersion involves mixing CNTs uniformly in a polymer via non-covalent interactions (Ding et al., 2024). However, CNTs tend to aggregate, so dispersants or structure-directing agents (such as clay nanosheets or emulsifiers) are often needed. Surface functionalization improves the CNTs’ hydrophilicity and biocompatibility through covalent or non-covalent modifications (Deng et al., 2022; Ravanbakhsh et al., 2020). For example, oxidative treatment can introduce hydroxyl or carboxyl groups at the ends of CNTs, enabling them to form cross-links or hydrogen bonds with the hydrogel matrix (Forero-Doria et al., 2020).

Carbon nanomaterials are photothermally stable with excellent thermal conductivity and large surface areas for drug loading, and they can be surface-tailored to improve dispersibility and biocompatibility. On the other hand, pristine carbon agents are intrinsically hydrophobic and not readily biodegradable-they tend to aggregate in biological media and may persist in tissues, so appropriate functionalization or nanoscale design is required to mitigate potential chronic toxicity (Wang P. et al., 2025).

### 2.2 Organic photothermal materials for mild photothermal hydrogels

Compared to traditional inorganic materials, organic photothermal agents generally have better biocompatibility, making them suitable for long-term implantation *in vivo*. Their molecular structures are highly tunable, allowing researchers to adjust their optical absorption peaks via molecular design and functional modification to match the “NIR biological window” (∼650–950 nm for NIR-I; ∼1,000–1,350 nm for NIR-II), where absorption by hemoglobin and water is minimal, allowing deeper tissue penetration and thus improving the efficacy of photothermal tumor ablation (Guo S. et al., 2023). Typical organic photothermal agents include conductive polymers, and organic dyes (Makabenta et al., 2021; Wu et al., 2023).

#### 2.2.1 Conductive polymer photothermal agents

Conjugated conductive polymers such as polypyrrole (PPy), polyaniline (PANI), and poly (3,4-ethylenedioxythiophene) (PEDOT) usually have broad absorption in the near-infrared region. These materials also possess electrical conductivity and thermal stability, and they easily integrate with hydrogel networks to form composites. Polypyrrole (PPy), due to its dark conjugated structure, exhibits excellent photothermal performance. One study constructed a three-dimensional porous hydrogel containing PPy and loaded with a heat-sensitive nitric oxide donor (BNN6). After 10 min of 808 nm laser irradiation (1.0 W/cm^2^), the PPy component achieved an approximately 80% photothermal conversion efficiency and triggered the release of NO from the donor, combining mild photothermal effects with chemical antimicrobial action (Guo W. et al., 2023). Polyaniline (PANI) is another typical conductive polymer photothermal agent. Researchers have prepared methacrylate-terminated polyaniline nanoparticles (Me-PANI NPs) and used the vinyl groups as chemical crosslinking points to construct a PANI-crosslinked conductive photothermal hydrogel. *In vitro* experiments showed that the introduction of Me-PANI NPs enabled the hydrogel to exhibit mild photothermal antibacterial activity under NIR irradiation, while also endowing the hydrogel with excellent mechanical properties (Pang et al., 2022).

Conjugated polymers exhibit broad NIR absorption and can achieve high photothermal efficiencies while generally showing good biocompatibility, importantly, many can be engineered to be biodegradable, addressing long-term safety to a degree. A key limitation, however, is that maintaining optimal photothermal performance and assured biodegradability can be challenging-polymer nanostructure and doping chemistry influence stability and heat conversion, and incomplete degradation or byproducts could still pose biocompatibility issues that require careful design (Bao et al., 2025).

#### 2.2.2 Organic dye photothermal agents

Organic dye photothermal agents include both naturally derived pigments and synthetic small-molecule dyes that strongly absorb NIR light. Compared to inorganic nanomaterials, these organic agents generally exhibit superior biocompatibility and avoid heavy-metal-associated toxicities (Zhou et al., 2025). Natural pigments (e.g., phycocyanin, a blue phycobiliprotein from Spirulina) offer excellent biocompatibility and even possess inherent bioactive properties (immune-regulatory, antioxidant, anti-inflammatory) (Bai et al., 2023). However, as proteinaceous pigments they are prone to degradation under heat or light, which can reduce photothermal stability. This limits their standalone photothermal efficacy, and thus they are often combined with nanomaterials to enhance light-to-heat conversion. Synthetic dyes (e.g., indocyanine green (ICG) and other indocyanines like IR780/IR820) provide high molar absorption in the NIR and effective photothermal conversion. Indeed, ICG is an FDA-approved imaging agent that has also been widely explored as a photothermal agent (Cai et al., 2025). Their key drawback is instability in physiological conditions - ICG and its analogues tend to photobleach, aggregate, and clear rapidly from the body. Moreover, certain dyes, such as IR780 and IR820, are highly hydrophobic, necessitating encapsulation in carriers (polymers, liposomes, proteins, etc.) to improve water dispersibility and stability (Fialho et al., 2025). Such methods have been shown to prevent dye aggregation/photobleaching and prolong circulation, thereby significantly improving the photothermal performance of these organic dye agents.

Generally speaking, organic dye photothermal agents are typically low-cost, biocompatible, and readily excreted, with strong NIR absorption profiles, but they generally have lower photothermal conversion efficiency and limited photostability (e.g., photobleaching and rapid clearance), constraining their effectiveness in sustained photothermal therapy (Teng et al., 2024).

### 2.3 Photothermal agent loading strategies in hydrogels

Constructing a stable, efficient, and responsive mild photothermal hydrogel system is closely related to the method of incorporating photothermal agents into the hydrogel. Common loading strategies include: 1) Physical encapsulation: this method directly embeds photothermal agents into the hydrogel network through electrostatic adsorption, hydrophobic interactions, or van der Waals forces (Hu et al., 2024). The construction process is simple and requires mild reaction conditions. For example, GO or CuS nanoparticles can be mixed with sodium alginate, gelatin, etc., and crosslinked with Ca^2+^ or glutaraldehyde to form composite hydrogels (Huang et al., 2023). Physical encapsulation is straightforward, but lacking covalent bonds, the photothermal agents may “leak” or migrate over time with prolonged use. 2) Covalent conjugation: specific chemical reactions are used to covalently bind photothermal agents to the hydrogel network, enhancing system stability and sustained responsiveness. For instance, GO bearing surface carboxyl groups can be grafted onto an amino–functionalized gelatin backbone via EDC/NHS coupling, or polymer–photothermal agent covalent networks can be built through thiol–ene “click” chemistry (Degirmenci et al., 2024; Zhang W. et al., 2025). 3) *In situ* self–assembly: a newer strategy is to form a three-dimensional network via the self–assembly of the photothermal agent itself or its non-covalent interactions with polymers. For example, ICG and a gelatin/PEG matrix can be co–assembled into a self-healing photothermal hydrogel via a freeze–dry–rehydration approach, yielding a material with responsiveness, moldability, and biodegradability (Li et al., 2022). In addition, certain natural small molecules (such as tannic acid) have dual abilities to chelate metal ions and stack π–π bonds, allowing them to induce gelation *in situ* under mild conditions, which is an important direction for green self-assembly strategies (Zhou et al., 2024) (Figure 1b).

## 3 Biomedical applications of mild photothermal hydrogels

### 3.1 Applications in wound healing

**FIGURE 2:**
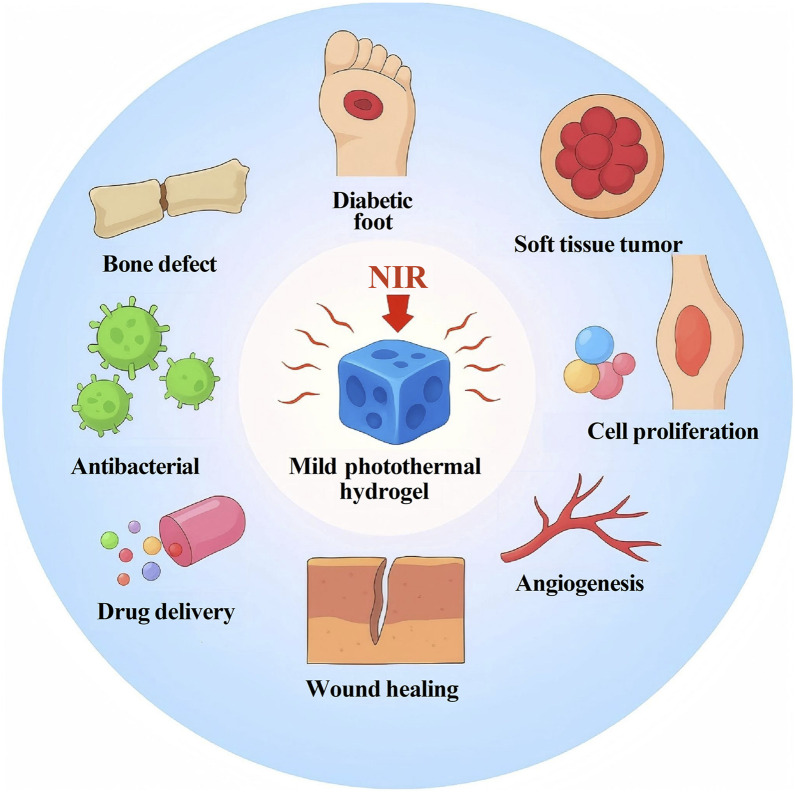
Current main biomedical applications of mild photothermal hydrogels.

Both chronic and acute wounds are often accompanied by pathological changes such as disruption of the skin barrier, persistent infection, high oxidative stress, and chronic inflammation, which make healing difficult. There is an urgent need for novel multifunctional therapeutic strategies to address these multiple challenges simultaneously (Byun et al., 2024). In recent years, many researchers have achieved significant results in combating infection and controlling pathogens in wounds by combining mild photothermal effects with multimodal antibacterial strategies (Figure 2). For example, Gao et al. prepared a chitosan hydrogel containing ZIF–8 nanoparticles coated with polydopamine (PDA). Under 808 nm laser irradiation, the temperature of this hydrogel was maintained at 40 °C–45 °C. This system, through Zn^2+^-mediated bacterial membrane rupture combined with local mild heating, was highly effective against methicillin-resistant *S. aureus* (MRSA), achieving a 99.5% kill rate (Gao et al., 2025). In a burn infection model, Yu et al. prepared a gelatin–oxidized dandelion hydrogel containing natural black currant extract. Under 808 nm irradiation (2.5 W/cm^2^, temperature controlled at 45 °C), 10 min of treatment could kill 99% of *S. aureus*, 98% of *E. coli*, and 82% of *P. aeruginosa* (Yu et al., 2024). Mild photothermal therapy (PTT <45 °C) exerts broad antibacterial effects through multiple mechanisms. First, sub-lethal photothermal heating damages bacterial membranes, increasing their permeability and causing leakage of cytoplasmic contents, which leads to cell lysis (Zhang et al., 2022). Second, heat stress can denature bacterial proteins (including enzymes and structural proteins), impairing essential cellular functions. Third, mild PTT may elevate bacterial reactive oxygen species (ROS) levels, inducing oxidative damage to lipids, proteins, and DNA within the microbes (Chen et al., 2025). Fourth, localized photothermal heating can disrupt biofilm structures by weakening the extracellular polymeric matrix and enhancing the susceptibility of biofilm-encased bacteria to treatment. Finally, mild hyperthermia can modulate the host immune response at the wound site–for example, by promoting immune cell recruitment and activation, which aids in clearing the infection (Zhao et al., 2023). Collectively, these mechanisms enable mild photothermal therapy to effectively reduce bacterial burden and facilitate wound healing while minimizing damage to healthy tissue.

Beyond efficient antibacterial activity, using the inherent biological activities of materials to correct the wound microenvironment’s oxidative stress and chronic inflammation is equally crucial. Ma et al. reported a composite hydrogel containing black phosphorus (BP) nanosheets and quaternized chitosan (QCS). Upon NIR irradiation, this hydrogel significantly reduced the expression of pro-inflammatory cytokine IL-6 and increased the expression of anti-inflammatory IL-10. This anti-inflammatory effect is attributed to the synergistic action of QCS itself and the mild photothermal stimulation mediated by BP (Ma et al., 2025). Li et al. used dopamine-modified hyaluronic acid and PDA-coated Ti_3_C_2_ MXene nanosheets to prepare an injectable, self-catalyzing cross-linked hydrogel. Leveraging the non-enzymatic antioxidant properties of the MXene photothermal agent, this system efficiently scavenges reactive oxygen species (ROS) and maintains cellular redox homeostasis (Li et al., 2022). Simultaneously, the HA-DA (hyaluronic acid-dopamine) scaffold induces macrophages to polarize toward the anti-inflammatory M2 phenotype. Together, these effects allow the hydrogel to effectively improve the pathological microenvironment of infected diabetic wounds.

Adequate angiogenesis is also essential for wound healing. Huang et al. reported a copper/Zn-MOF composite hydrogel with photothermal functionality. This hydrogel precisely modulated the M1/M2 balance of macrophages at the wound site, skewing macrophages toward the M2 phenotype, thereby promoting neural tissue and blood vessel regeneration and accelerating chronic wound healing (Huang K. et al., 2022). Gao et al. constructed a tri-component crosslinked hydrogel made of carboxymethyl chitosan (CMCS), gelatin, and oxidized sodium alginate (OSA), into which PDA-modified ZIF-8 nanoparticles were embedded. Under mild NIR irradiation, this material promoted endothelial cell proliferation and the expression of angiogenic markers (VEGF and CD31), enhancing blood vessel formation and collagen alignment, which together accelerated wound closure (Gao et al., 2025). In addition, other studies have incorporated growth factors (such as VEGF and bFGF) or nanoparticles (such as Au nanoparticles or PbS quantum dots) into mild photothermal hydrogels (Guo et al., 2024; Xia et al., 2021; Zhu et al., 2023). The mild heating from NIR irradiation stimulates controlled release of these factors, and combining the system with immunomodulatory molecules (such as IL-10 or TGF-β) can further enhance angiogenesis and immune-mediated healing.

### 3.2 Applications in bone and soft tissue tumor therapy

Conventional tumor treatments (including surgical resection and radiotherapy) often face issues such as residual tumor cells post-surgery, local recurrence, cancer cell drug resistance, and systemic toxicity from high-dose chemotherapy. High-temperature photothermal therapy has been shown to directly lyse cancer cells, but excessively high temperatures (>50 °C) can cause damage to normal tissues and elicit inflammatory responses (Xia et al., 2021). Therefore, a series of recent studies have focused on designing and applying mild photothermal systems (Figure 2). Luo et al. reported an HTA hydrogel constructed from hydroxypropyl chitosan, tannic acid, and Fe^3+^ complexes. Under 808 nm laser irradiation (1 W/cm^2^), the hydrogel temperature stabilized at 42 °C–43 °C, significantly inducing apoptosis in osteosarcoma cells and causing tumor-associated macrophages to shift from the M2-type to the M1-type phenotype (Luo et al., 2023). This confirmed that a mild photothermal effect can achieve both immune microenvironment remodeling and antitumor effects. Chen et al. developed a composite hydrogel of silk fibroin, sericin-dopamine, tannic acid, and Cu^2+^. Under 808 nm laser irradiation (0.75 W/cm^2^, 20 min), the hydrogel temperature was maintained at approximately 44 °C; it was able to inhibit the proliferation of osteosarcoma cells and induce apoptosis, while the Cu^2+^ and polyphenols exerted antioxidant effects that improved the tissue repair environment (Chen et al., 2025). In another study, Zhang et al. developed an injectable gelatin-dopamine hydrogel composite with magnesium peroxide (MgO_2_) nanoparticles. Under 808 nm laser irradiation (0.5 W/cm^2^, 10 min), the hydrogel temperature reached about 43 °C. This hydrogel not only suppressed osteosarcoma cell activity through the mild photothermal effect but also released Mg^2+^ to promote bone regeneration, demonstrating a dual “antitumor and osteogenic repair” function (Zhang R. et al., 2025). These results indicate that by precisely adjusting the concentration of photothermal agents and the irradiation conditions, hydrogels can stably generate a mild heating effect. This enables dual therapeutic effects of antitumor activity and tissue repair without damaging normal tissues.

Mild photothermal therapy (heating tissues to <45 °C) can itself impede tumor growth via multiple mechanisms. Even moderate hyperthermia (≈42 °C–45 °C) induces partial protein denaturation, membrane disruption, and oxidative stress in cancer cells. These sub-lethal injuries trigger mitochondrial dysfunction-elevated temperature increases mitochondrial membrane permeability and ROS generation-which in turn activates intrinsic apoptosis pathways (Cai et al., 2025). Consequently, mild photothermal heating can promote tumor cell apoptosis without reaching ablative temperatures (Huang Q. et al., 2022). On the other hand, many hydrogel-based systems combine mild photothermal effects with additional therapies to achieve synergistic antitumor activity. The photothermal agents (e.g., polydopamine, gold nanomaterials) embedded in the hydrogel convert NIR into heat, raising the local temperature above a critical transition. This can induce a volume phase change in thermo-responsive polymers or break thermosensitive linkages, freeing the payload. For example, Liu et al. reported a chitosan-based hydrogel that remains stable at 37 °C but, upon NIR irradiation, undergoes rapid network collapse into a porous state, thereby releasing its drug load on-demand (Liu et al., 2021). Likewise, Kong et al. designed an injectable liposomal hydrogel in which an NIR photothermal dye generates mild heat to rupture encapsulated thermosensitive liposomes, instantly releasing gemcitabine at the target site. Immune checkpoint inhibitors (such as anti-PD-1/PD-L1 antibodies) or cytokines (e.g., IFN-γ, IL-12) can be co-encapsulated in injectable hydrogels for sustained, localized delivery to the tumor (Kong et al., 2021). The hydrogels protect these biomolecules and concentrate them in the tumor microenvironment, improving therapeutic index (Mohammadzadeh et al., 2025). Mild photothermal therapy (sub-ablative hyperthermia) further enhances their efficacy by stimulating immunogenic tumor cell death and promoting immune cell infiltration. For instance, a recent alginate hydrogel loaded with anti-PD-L1 antibodies and Fe_3_O_4_ nanoparticles showed that NIR irradiation induced tumor cell apoptosis and the release of tumor antigens, while simultaneously releasing anti-PD-L1 *in situ*; this led to robust T-cell activation and tumor regression *in vivo* compared to antibody or PTT alone (Mohammadzadeh et al., 2025). In general, the heat from mild PTT can “prime” tumors for immunotherapy-increasing dendritic cell maturation and cytotoxic T-lymphocyte activity and upregulating checkpoint ligand expression on cancer cells-thereby transforming an immunosuppressive tumor into an “immunologically hot” state more responsive to checkpoint blockade therapy.

### 3.3 Applications in bone defect repair

Like wound healing, the treatment of bone defects faces many challenges. Firstly, due to limited donor availability, autograft bone transplantation is constrained and can lead to immune rejection and complications. In addition, bone infection—especially that caused by drug-resistant bacteria secondary to trauma or surgery—often complicates treatment. Bone defect and infection sites also often suffer from poor circulation, hypoxia, and an excessive local immune response (Wu A. L. et al., 2025).

Studies have shown that mild heat stimulation (in the range of 37 °C–45 °C) can upregulate heat shock proteins (e.g., HSP70, HSP90) and indirectly or directly activate osteogenic signaling pathways such as Wnt/β-catenin, PI3K/AKT/mTOR, BMP/Smad, and MAPK (ERK1/2), thereby promoting the expression of bone markers (ALP, OCN, COL-I) (Yao et al., 2024) (Figure 2). Wang et al. reported a photothermal hydrogel system based on silk and calcium phosphate composites. By near-infrared (NIR) irradiation (808 nm, 1 W/cm^2^ for 5 min), the local temperature was raised to 42 °C. The mild photothermal effect activated heat shock proteins and osteogenesis-related genes (Runx2 and ALP), promoting the proliferation and osteogenic differentiation of bone marrow mesenchymal stem cells (BMSCs) (Wang et al., 2023). Li et al. designed a photothermal hydrogel system containing gold nanoparticles; using 808 nm NIR light (1 W/cm^2^ for 6 min), they generated a mild photothermal effect (∼40 °C) in the hydrogel. This promoted the osteogenic differentiation of BMSCs and also enhanced bone mineralization by releasing dissolved calcium ions (Li X. et al., 2023).

In addition, antibacterial activity, immune regulation, and pro-angiogenic effects are also considered crucial in bone defect treatment. Wei et al. combined copper nanoparticles with a photothermal hydrogel; under NIR irradiation (808 nm, 1.5 W/cm^2^, 5 min), the copper nanomaterials and the mild heat effectively killed bacteria in a bone infection model, accelerating the healing of the bone defect (Wei et al., 2024). Sun et al. designed a photothermal hydrogel system using 808 nm NIR laser irradiation (1 W/cm^2^, 5 min) to achieve a mild photothermal effect that raised the local temperature to 42 °C. This thermal stimulation not only promoted polarization of macrophages to the M2 anti-inflammatory phenotype, reducing inflammation, but also increased the expression of angiogenic factors such as VEGF and bFGF, promoting new blood vessel formation (Sun et al., 2025).

## 4 Concluding and perspectives

To date, researchers have successfully constructed various composite hydrogels with mild photothermal functionality and have achieved precise control over temperature elevation in space and time. However, several challenges and bottlenecks remain. Technically, the shallow penetration of NIR light in tissue (typically only a few millimeters to ∼1 cm) limits the treatment of deep lesions. Furthermore, current platforms lack real-time thermal feedback mechanisms, making it difficult to precisely control the local temperature during therapy; the long-term safety of repeated NIR irradiations also remains to be fully evaluated. A delicate balance must be struck between photothermal efficacy and biocompatibility-high laser powers or photothermal agent doses can produce effective heating but may injure surrounding healthy tissue. Translationally, there are substantial hurdles in scaling up the production of photothermal hydrogel systems while maintaining reproducibility and regulatory compliance, which complicates their path to clinical use. Indeed, despite encouraging preclinical outcomes, most reported mild photothermal hydrogel therapies have yet to progress into clinical trials, highlighting the gap between laboratory research and clinical translation (Sun et al., 2024).

To address these issues, future improvements could include: 1) Developing new NIR-II responsive materials: enhancing tissue penetration by using materials activated by the second near-infrared window. 2) Introducing self-regulating thermal elements or degradable thermal buffering layers: Achieving a smoother and more stable heat output. 3) Exploring combinations with non-optical stimulation methods: Incorporating stimuli such as ultrasound or electrical stimulation, which can penetrate deeper into tissues, to complement photothermal therapy and overcome the limitations of light penetration (Xie et al., 2022; Zhu et al., 2024).

In summary, as a biomedical material with immense potential, mild photothermal hydrogels are poised to play an increasingly important role in future tissue repair and regeneration. Through interdisciplinary collaboration and continuous innovation, we can anticipate that mild photothermal hydrogels will offer patients more effective, safer, and more personalized treatment options.
